# Supplementary material for: SLC25A46 is required for mitochondrial lipid homeostasis and cristae maintenance and is responsible for Leigh syndrome
Source: EMBO Mol Med. 2016 Jul 7;8(9):1019–38. doi: 10.15252/emmm.201506159 (PMC5009808; doi:10.15252/emmm.201506159)
Supplement: Supplementary file 1 — Appendix [file EMMM-8-1019-s001.pdf]

Appendix

Table of Contents

1. Clinical history of the subject
2. Table S1. SLC25A46 proteins in the EMC complex identified in the BioID assay.
3. Table S2. Mass spectrometry analysis of mitochondrial phospholipid content in mitochondria isolated from control and subject fibroblasts.

Clinical History of the subject

The pregnancy was marked by gestational diabetes treated with insulin, and by mild oligohydramnios. Birth was by spontaneous vaginal delivery at term. Meconial staining of amniotic fluid was noted. Birth weight was 2.55 kg (3rd percentile), length 48 cm (15-50%) and head circumference 34 cm (50%). Apgar scores were 6, 7 and 8 at 1, 5 and 10 minutes. The initial course was normal but at 4 hours of age she had a convulsion lasting for 20 seconds, which did not recur. An electrocardiogram and a chest radiogram were normal. Electroencephalography and cerebral CT scanning were normal. She was discharged after two days.

She was irritable child, with feeding difficulties suggesting gastroesophageal reflux. At 4 months of age, a pediatric neurologist noted constant developmental progress, irritability and difficulty sleeping at night. Examination revealed a weight of 5.8 kg (15%), length 58 cm (3%), head circumference 41.5 cm (55%) and mild hypertonia.

She was referred again at age 8.5 months for psychomotor delay and growth failure. She was thin and had mild spastic diplegia, but was alert and interacted appropriately with her surroundings. An ophthalmologist noted intermittent esotropia.

Laboratory assessment on admission included blood pH 7.43, pCO2 26.3 (normal: 34-45), bicarbonate 17 mmol/L (normal: 21-25), lactate 2.19 mmol/L (normal: 0.5-2.2), pyruvate 55 µmol/L (normal: 34-190), plasma sodium 140 mmol/L (normal: 130-142), potassium 4.7 mmol/L (normal: 3.6-6.2), chlorine 112 mmol/L (normal: 103-110). Plasma amino acid chromatography was normal, including alanine 274 µmol/L (normal: 182-396). Lumbar puncture revealed lactate 2.56 mmol/L (normal: 1.10-2.20) and pyruvate 136 µmol/L (normal: 34-125).

The subject had hypercalciuria but urinary amino acid excretion was normal and glucosuria was absent. She was treated with sodium bicarbonate. A fluoroscopic study of swallowing revealed a slow and somewhat uncoordinated oral phase, with nasopharygeal reflux of clear liquids and gagging with solids. A cerebral magnetic resonance (MRI) study was normal. Karyotype and an abdominal ultrasound were normal. At 11 months she was alert, had good ocular pursuit and improved head control but had definite motor delay.

At 13.5 months of age she was hospitalized for low-grade fever and vomiting. Two weeks later she was transferred to CHU Sainte-Justine. Soon after transfer, fever (39.5°C) and marked diaphoresis were noted. The patient had a convulsion. Sinus tachycardia (215/minute) and gasping respirations were noted. She was intubated. Cerebral computerized tomography showed multiple lesions in the cerebellar white matter, cerebellar peduncles and brain stem. Cerebral magnetic resonance imaging (MRI) confirmed lesions in the cerebellar hemispheres and periaqueductal grey matter, with mild involvement of the globi pallidi. These lesions were hypointense in T1-weighted and hyperintense in T2-weighted images. During this period, normal values were repeatedly obtained for lactate (1.87 ± 0.22 mmol/L, mean ± SEM, n=8; range, 1.10-3.08) and pyruvate (79.6 ± 8.9 µmol/L, mean ± SEM, n=8; range 30-101). On histological examination, a muscle biopsy showed neurogenic atrophy and an absence of ragged red fibers. Liver biopsy morphology was normal.

The subject remained ventilator-dependent. Although she could spontaneously open her eyes, she showed no interaction with her surroundings. A neuro-ophthalmologic consultant reported pallor of the optic disks bilaterally. The maculae appeared normal. There was bilateral internuclear ophthalmoplegia, suggestive of involvement of the medial lateral fasciculus. A neurological consultant found hyperreflexia and mild spasticity. Electromyography and nerve conduction velocity testing were not performed. One month later, repeat cerebral MRI showed marked progression of lesions in the brain stem and midbrain, including the medial lateral fasciculus, and abnormalities of the thalami. The parents elected to stop life support measures and the patient died soon thereafter. Autopsy was declined.

During this patient's course, systemic signs and signs in organs other than brain were subtle or absent. The subject had mild, intermittent acidosis. Lactate and pyruvate were marginally elevated in the cerebrospinal fluid but not in blood. The uncoordinated swallowing, breathing abnormalities and the reduced level of consciousness that dominated the final stage of her course, and possibly the fever of unknown origin and tachycardia, can be explained by brain stem dysfunction. Together, the neurological signs, chronology of clinical events and the location and type of cerebral imaging abnormalities are diagnostic for Leigh Syndrome.

Only a small number of *SLC25A46*-deficient patients have been reported to date and our patient extends the clinical spectrum to include an important clinical presentation of mitochondrial disease, Leigh syndrome. The patient shows both similarities and important differences to the previous report. The combination of childhood-onset optic atrophy and hyperreflexia or spasticity in young adulthood recurred in different *SLC25A46*-deficient families. Our patient had pale optic disks, perhaps reflecting early optic atrophy, and had active deep tendon reflexes. These findings were minor compared with the patient's other neurological signs, and detailed testing of peripheral nerve and optic nerve function was not performed. It is possible that peripheral neuropathy or optic atrophy may have developed had the patient survived longer. Basal ganglion calcification, a feature of Leigh disease and of other mitochondrial disorders, was reported in a 44-year-old *SLC25A46*-deficient patient (Family IT), and our patient had abnormalities of the globi pallidi on MRI imaging at one year of age. In our patient, clinical signs were dominated by neurological signs related to abnormalities of the deep grey matter of the hindbrain. On cerebral MRI, these lesions had the typical distribution, symmetry and T2-weighed hyperintense, T1-weighted hypointense characteristics of Leigh disease. Leigh disease was not previously diagnosed in *SLC25A46*-deficient patients. However, the patient of Family US was abnormal at birth, showed progressive atrophy of the cerebellum and brainstem and required endotracheal intubation and respiratory support. Although the diagnosis of Leigh syndrome was not mentioned in relation to that patient, these clinical signs are compatible.

	SLC25A46 partner proteins identified in bio ID			
	alternative name	yeast homologue	number of spectra	fold change
**EMC1**	KIAA0090	YCL045C-EMC1	33	88
**EMC2**	KIAA0103-TTC35	YJR088C-EMC2	9.5	76
**EMC3**	TMEM111	YKL207W-AIM27	7.5	60
**EMC4**	TMEM85	YGL231C-EMC4	2	20
**EMC5**	MMGT1-TMEM32	YIL027C	3.5	35
**EMC6**	TMEM93	YLL014W	2	10
**EMC7**	C15orf24		3	30
**EMC8**	FAM158B-COX4NB		3.5	35
**EMC9**	FAM158A		0.5	5

Supplementary table 2: analysis of mitochondrial lipid content by mass spectometry

				Normalized intensities with internal standards					
				control			subject		
				exp 1	exp 2	exp 3	exp 1	exp 2	exp 3
**Lipid class**	**Compound name**	**RT**	**Mass**						
**PA**	PA 36:4	20.60	714.507	60685	25834	32744	7903	5462	2898
	PA 38:5	20.50	740.522	172476	69934	101546	87481	56017	49057
	PA 40:6	20.74	766.538	17824	10463	14816	6292	7072	2857
**PC**	PC 28:0	17.86	677.5013	30288593	26750337	29454248	27253616	27893141	29263629
	PC 30:0	21.19	705.5322	7600828	3762276	5498484	4408347	2936940	3875674
	PC 32:0	24.90	733.5635	10236348	8787419	9064997	10683570	7675029	8432108
	PC 34:0	28.82	761.5942	2394438	1758069	2233349	3476974	2068990	3206507
	PC 34:1	25.55	759.5798	151614961	84752035	95515006	98149437	69581373	80620110
	PC 34:2-1	22.52	757.5635	16500000	8502535	9288359	4760373	3900839	3563773
	PC 34:2-2	22.84	757.5634	24407489	11609660	13747304	10497914	7598114	8364716
	PC 34:3-1	20.48	755.5464	2220103	1075898	915481	502061	603587	380020
	PC 34:3-2	21.40	755.5467	375942	70477	171871	388539	110714	256266
	PC 34:4	19.04	753.5311	195633	97687	92856	101759	79941	94540
	PC 36:0	32.90	789.6249	278488	259320	271950	315941	280791	308149
	PC 36:1-1	29.49	787.611	33098525	16524995	24100831	31766423	19647165	26072165
	PC 36:1-2	29.51	809.5912	4393687	2109442	3309092	4290579	2388156	3352801
	PC 36:2	26.18	785.5956	83796948	45686219	52805741	63001988	40596514	49310166
	PC 36:3-1	23.49	783.5785	8070360	5108161	4915669	2953936	2498469	2578074
	PC 36:3-2	23.97	783.5784	2823300	1698070	1659878	1467367	1588717	1380070
	PC 36:3-3	24.98	783.5785	8070360	5108161	4915669	2953936	2498469	2578074
	PC36:4-1	21.09	781.5626	344154	187250	196196	37754	55937	89781
	PC36:4-2	22.45	781.5626	2633207	3068697	1647858	2984177	2473769	1503811
	PC 36:5-1	19.67	779.5463	127367	205157	119007	127153	132203	166672
	PC 36:5-2	20.31	779.5467	662783	593050	396801	129909	307426	301575
	PC 38:0	35.83	817.6582	33397389	28955291	32360598	30042170	28704480	32833294
	PC 38:1	33.25	815.644	519732.7159	388808	399717	597470	473468	613009
	PC 38:2	29.89	813.6254	4150424	2437281	2942213	4016812	2838888	3599106
	PC 38:3-3	28.83	811.6094	695921	643283	411481	1294024	1570531	821461
	PC 38:3-1	27.01	811.6085	1840702	780384	1150261	1379398	909207	1150303
	PC 38:3-2	27.81	811.6094	695921	643283	411481	1294024	1570531	821461
	PC 38:4-1	24.62	809.5933	949957	783407	568661	592811	400601	479964
	PC 38:4-2	25.55	809.594	173770	119820	96127	467055	323035	272896
	PC 38:5	23.06	807.5784	2599013	2893778	1469453	3149151	3151787	1995513
	PC 38:6-1	20.87	805.5622	351349	341486	76103	174563	107682	116089
	PC 38:6-2	21.78	805.5618	731730	555350	390149	723371	523718	407320
	PC 40:4-1	28.43	837.625	243824	183208	142275	526339	385991	447548
	PC 40:4-2	29.02	837.6251	86407	74906	55147	346947	259501	251267
	PC 40:5	25.79	835.6092	335502	269107	270631	456452	416612	376287
	PC 40:5	26.60	835.609	602706	514019	322769	887301	597717	487930
	PC 40:6-1	23.47	833.5933	461897	403991	254528	428030	509816	388169
	PC 40:6-2	25.44	833.5936	457275	522769	258387	959547	752157	579259
	PC 40:7-1	22.37	831.5778	928694	684036	504382	902260	728516	692531
	PC 40:7-2	25.45	831.577	296816	346500	174828	700499	468727	413796
	PCO-34:0	31.22	747.6143	428564	386790	488182	1089299	534144	1026457
	PCO-34:1	27.68	745.5992	3499736	2843087	3267215	7891421	5625466	6978227
	PCO-34:2	24.97	743.5818	796712	482354	659312	939886	741939	1023581
	PCO-36:2	28.33	771.614	946858	400041	662468	1316121	1331859	1731729
	PCO-36:4	24.52	767.5825	552674	777093	447338	1676890	1361067	1307435
	PCO-38:3	27.57	819.6117	68230	69024	54741	351823	200977	212535
	PCO-38:4	28.42	795.6138	291273	292330	275687	906748	519504	588285
	PCO-38:5	25.04	793.5984	505490	710685	404405	2434663	1694874	1865241
	PCO-38:6	23.76	791.5822	154963	167483	116247	579783	298572	365233
	PCO-40:5	28.75	821.6287	95710	79015	83421	244871	124676	170325
**PE**	PE 32:0	25.60	690.508	14358	12205	12301	8737	8762	7567
	PE 32:1	22.58	689.5	160734	88882	87008	58086	50147	48279
	PE 34:1	26.30	717.5315	2597416	1220275	1484279	1117688	804460	631115
	PE 34:2	23.21	715.5168	876275	413793	526343	262417	287599	162814
	PE 36:0	30.25	747.578	305374	192071	187564	194706	153936	149926
	PE 36:1	30.27	745.5611	3584032	2394524	2443527	2459961	2767012	1829527
	PE 36:2	26.94	743.5473	5303111	2587362	3462088	2323298	1579121	1288591
	PE 36:3	24.20	741.5323	451459	199133	273698	94828	80197	132329
	PE 36:4	23.14	739.516	216363	160472	152323	141466	116958	72301
	PE 38:2	30.68	771.578	129252	100277	116226	97029	73320	74349
	PE 38:3	28.51	769.562	174590	115506	88671	52589	46874	47717
	PE 38:5	26.95	767.5454	1719228	1320374	1138245	2067334	1314448	946184
	PE 38:6	22.44	763.5194	165914	146691	79382	99286	92759	47951
	PE 40:6	26.16	791.5493	655732	445826	426914	565333	550095	460722
**PS**	PS 34:1	22.47	761.5197	463142	384863	315631	322942	367380	309782
	PS 34:2	19.70	758.498	32718	28963	23247	19234	14331	17003
	PS 36:1	23.05	788.543	166340	118985	100456	117853	95189	71152
	PS 36:0	23.69	791.5658	840928	413714	452531	391666	373276	383448
	PS 40:6	22.44	835.5345	349581	262252	231377	255077	251535	213744
