# Supplementary material for: SLC25A46 is required for mitochondrial lipid homeostasis and cristae maintenance and is responsible for Leigh syndrome
Source: EMBO Mol Med. 2016 Jul 7;8(9):1019–38. doi: 10.15252/emmm.201506159 (PMC5009808; doi:10.15252/emmm.201506159)
Supplement: Supplementary file 2 — Expanded View Figures PDF [file EMMM-8-1019-s002.pdf]

Expanded View Figures

PX(D,E)XX(K,R)

	PCD1	PCD2	PCD3
SLC25A1	$_{45}$PTEYVK$_{50}$	$_{142}$PMETIK$_{147}$	$_{240}$PLDVIK$_{245}$
SLC25A4	$_{28}$PIERVK$_{33}$	$_{133}$PLDFAR$_{138}$	$_{230}$PFDTVR$_{235}$
SLC25A7	$_{33}$PLDTAK$_{38}$	$_{133}$PTEVVK$_{138}$	$_{232}$PVDVVK$_{237}$
SLC25A46	$_{119}$PCIVLR$_{124}$	$_{215}$PFYSAS$_{220}$	$_{333}$PLETVL$_{338}$
MTCH1	$_{98}$PLLYVK$_{103}$	$_{214}$PLHVIS$_{219}$	$_{327}$PFLLVG$_{332}$
MTCH2	$_{20}$PLMYVK$_{25}$	$_{140}$PFHVIT$_{145}$	$_{236}$PFVLVS$_{241}$

Figure EV1. Sequences of the putative functional carrier (PCD) domains in three typical mitochondrial metabolite carrier proteins, SLC25A46, MTCH1, and MTCH2.
The consensus sequence is shown at the top of the figure. SLC25A1 is the citrate carrier, SLC25A4 is the adenine nucleotide translocase, and SLC25A7 is a proton carrier (uncoupling protein 1).

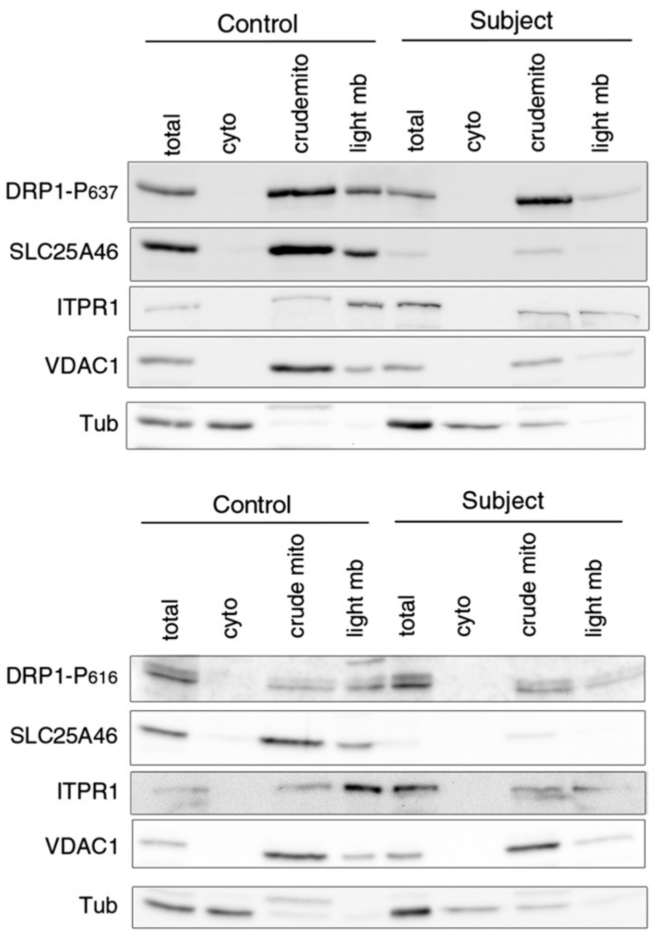

Figure EV2. Role of SLC25A46 in mitochondrial recruitment of phosphorylated forms of DRP1.
Immunoblot analysis of the localization of phosphorylated forms of DRP1 in control and subject fibroblasts after subcellular fractionation. Upper panel: DRP1 phosphorylated on serine 637. Bottom panel: DRP1 phosphorylated on serine 616. ITPR1 was used as an ER marker, VDAC1 as a mitochondrial marker, and alpha-tubulin as a cytosolic marker.

Source data are available online for this figure.

© 2016 The Authors
