# Supplementary figures and images for: SLC25A46 is required for mitochondrial lipid homeostasis and cristae maintenance and is responsible for Leigh syndrome
Source: EMBO Mol Med. 2016 Jul 7;8(9):1019–38. doi: 10.15252/emmm.201506159 (PMC5009808; doi:10.15252/emmm.201506159)

Figure EV2 raw data

Figure EV2, top

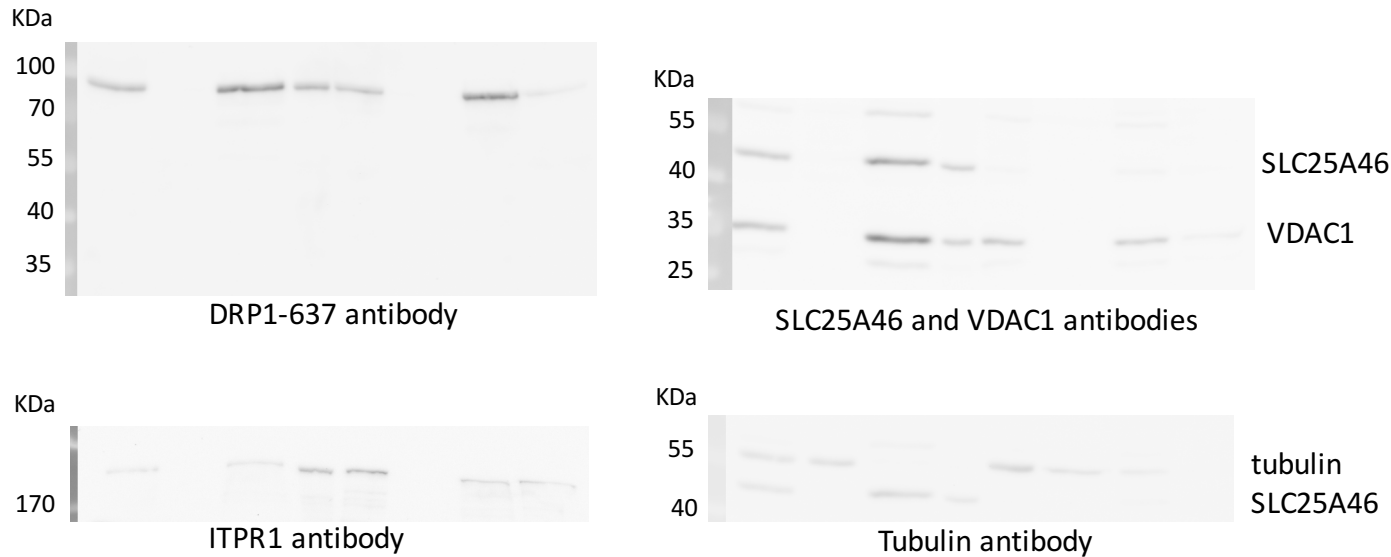

Figure EV2, bottom

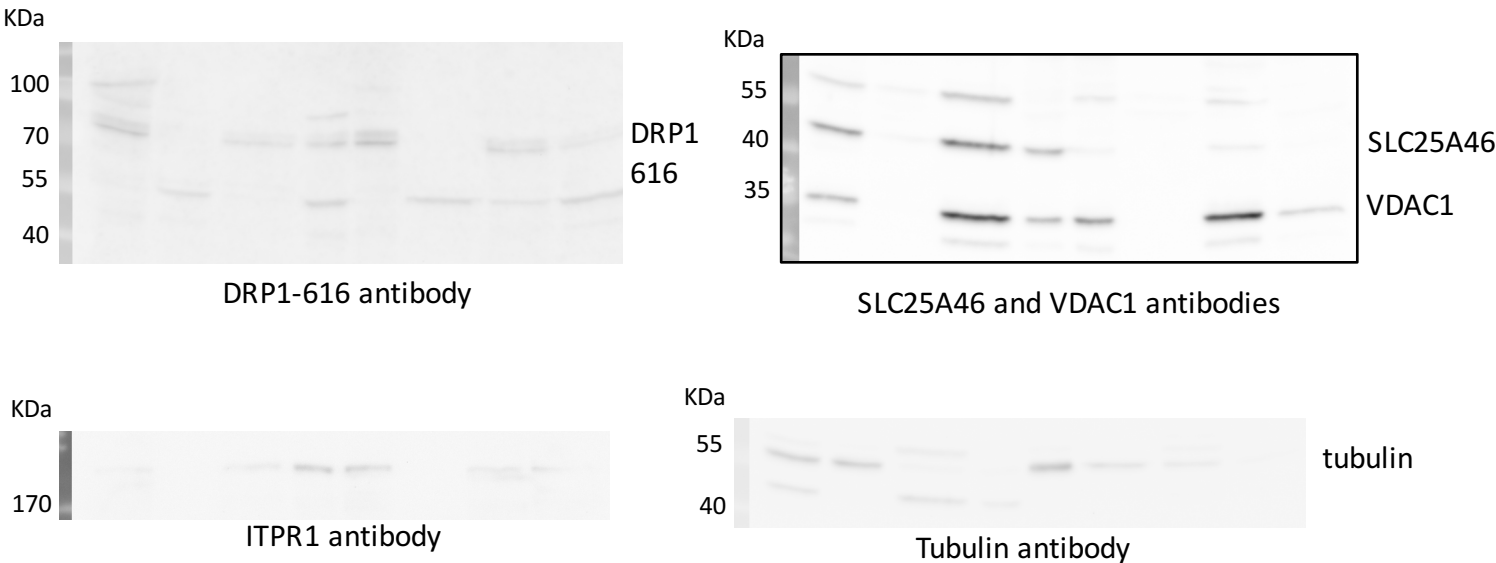

Supplement: Supplementary file 3 — Source Data for Expanded View [file EMMM-8-1019-s008.zip › Figure_EV2_raw_data.pdf]

Figure 1 raw data

Figure 1C

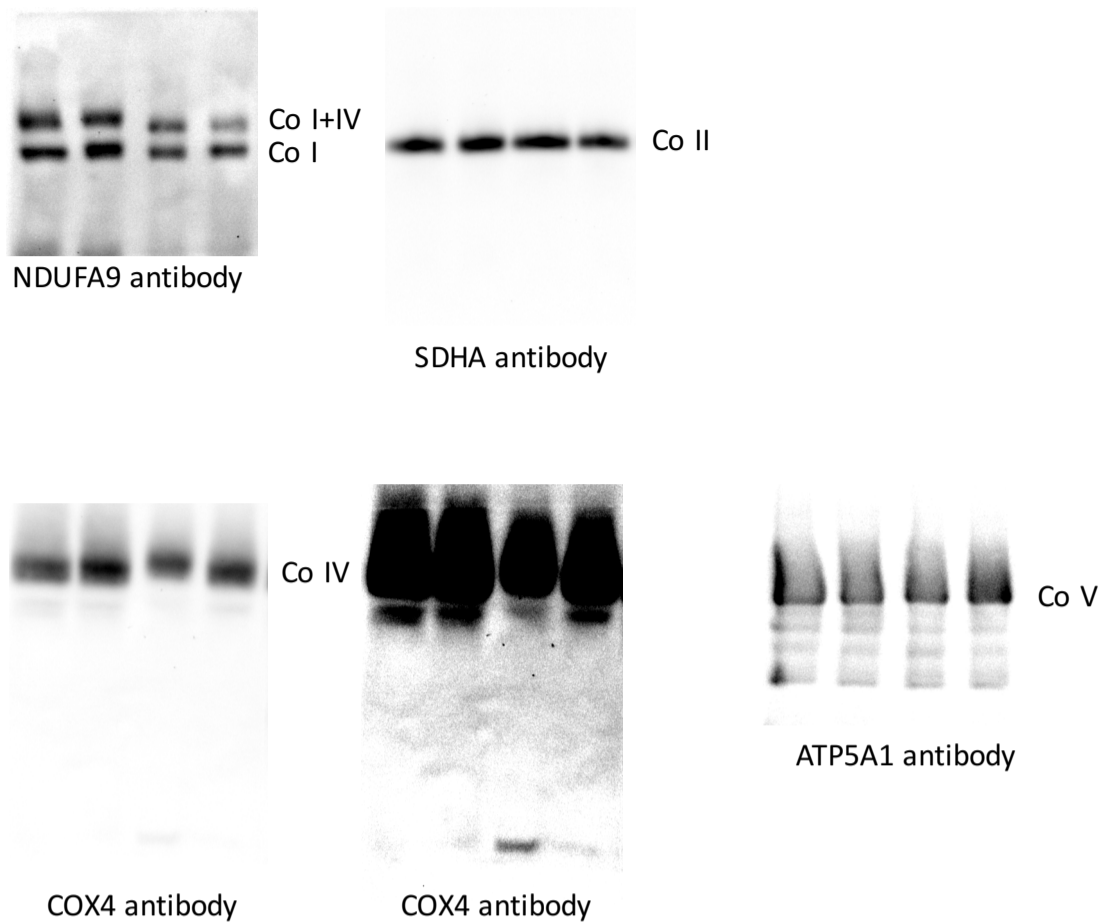

Figure 1D

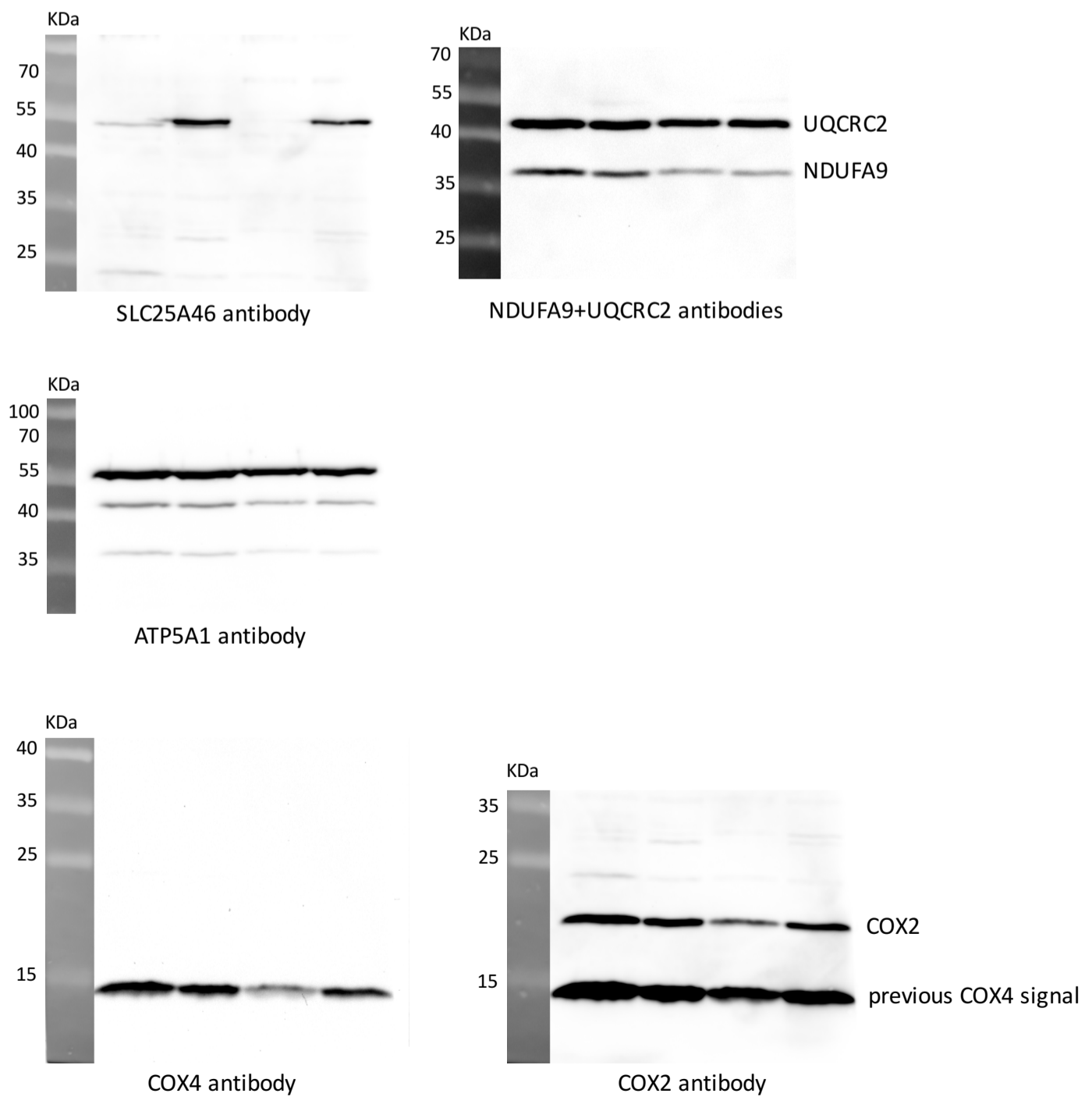

Supplement: Supplementary file 5 — Source Data for Figure 1 [file EMMM-8-1019-s003.pdf]

Figure 2 raw data

Figure 2A

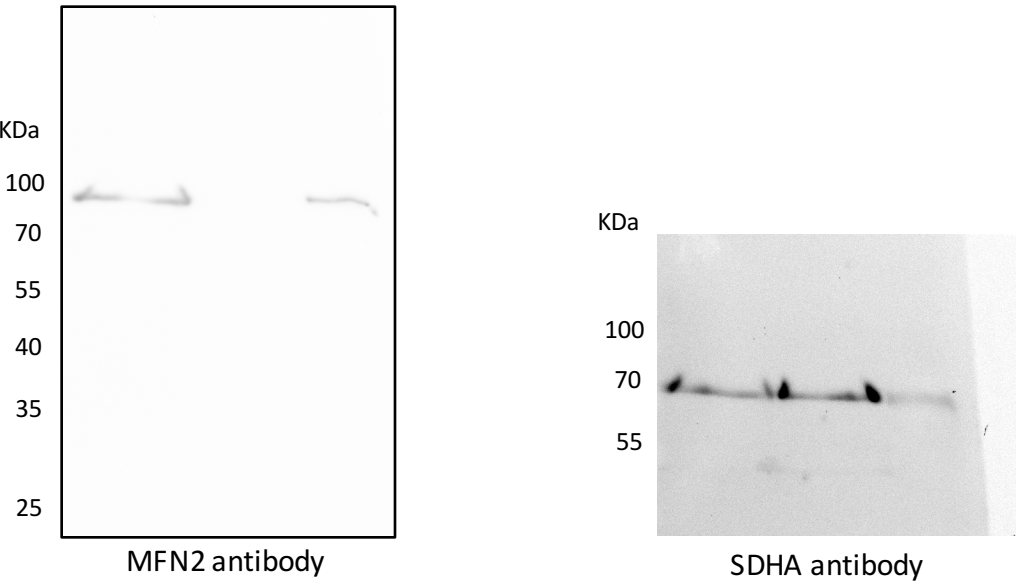

Figure 2B

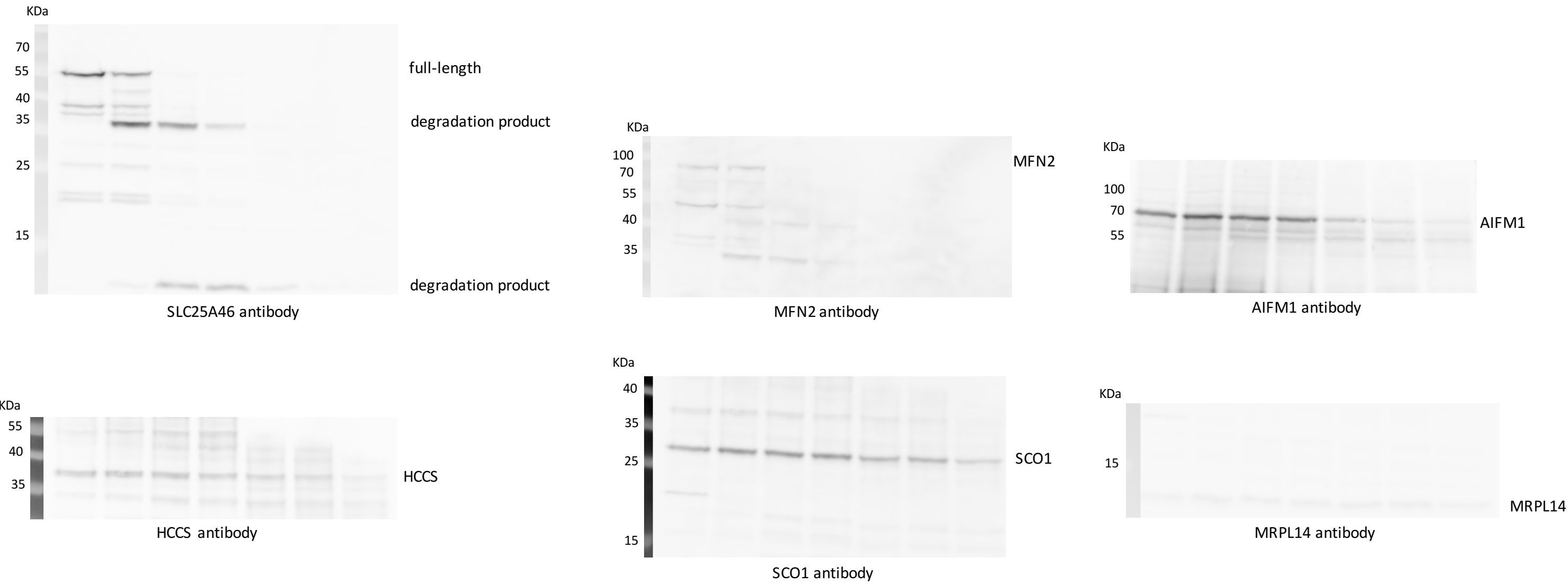

Figure 2C

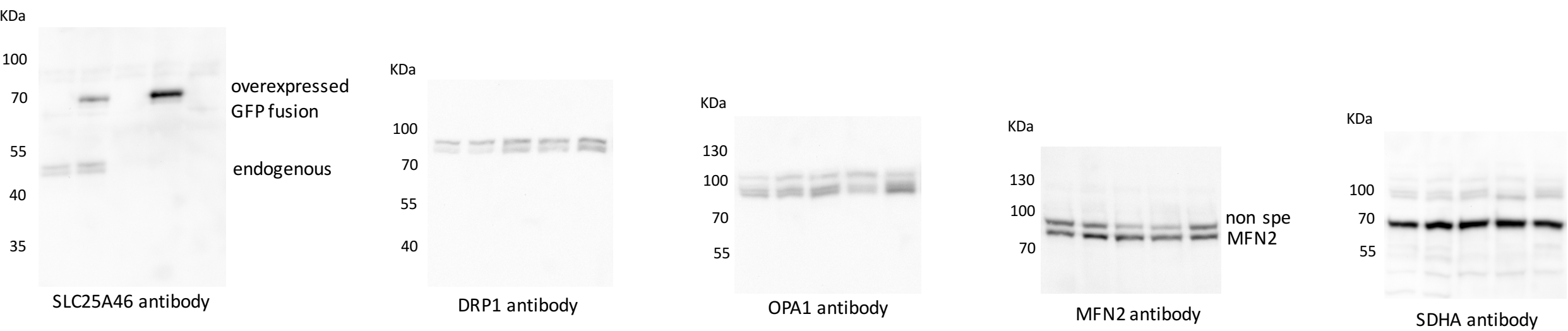

Supplement: Supplementary file 6 — Source Data for Figure 2 [file EMMM-8-1019-s004.pdf]

Figure 3 raw data

Figure 3C

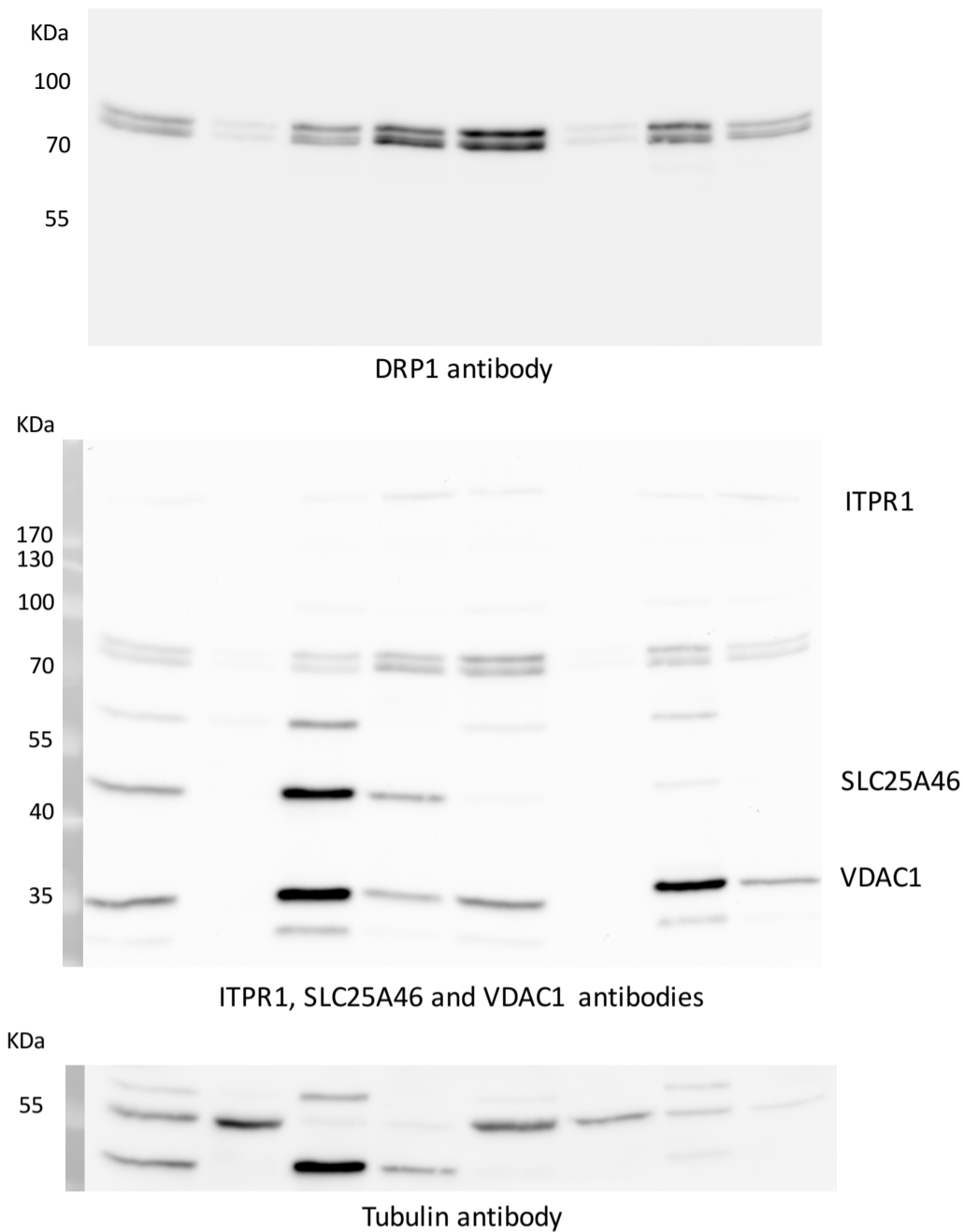

Figure 3D

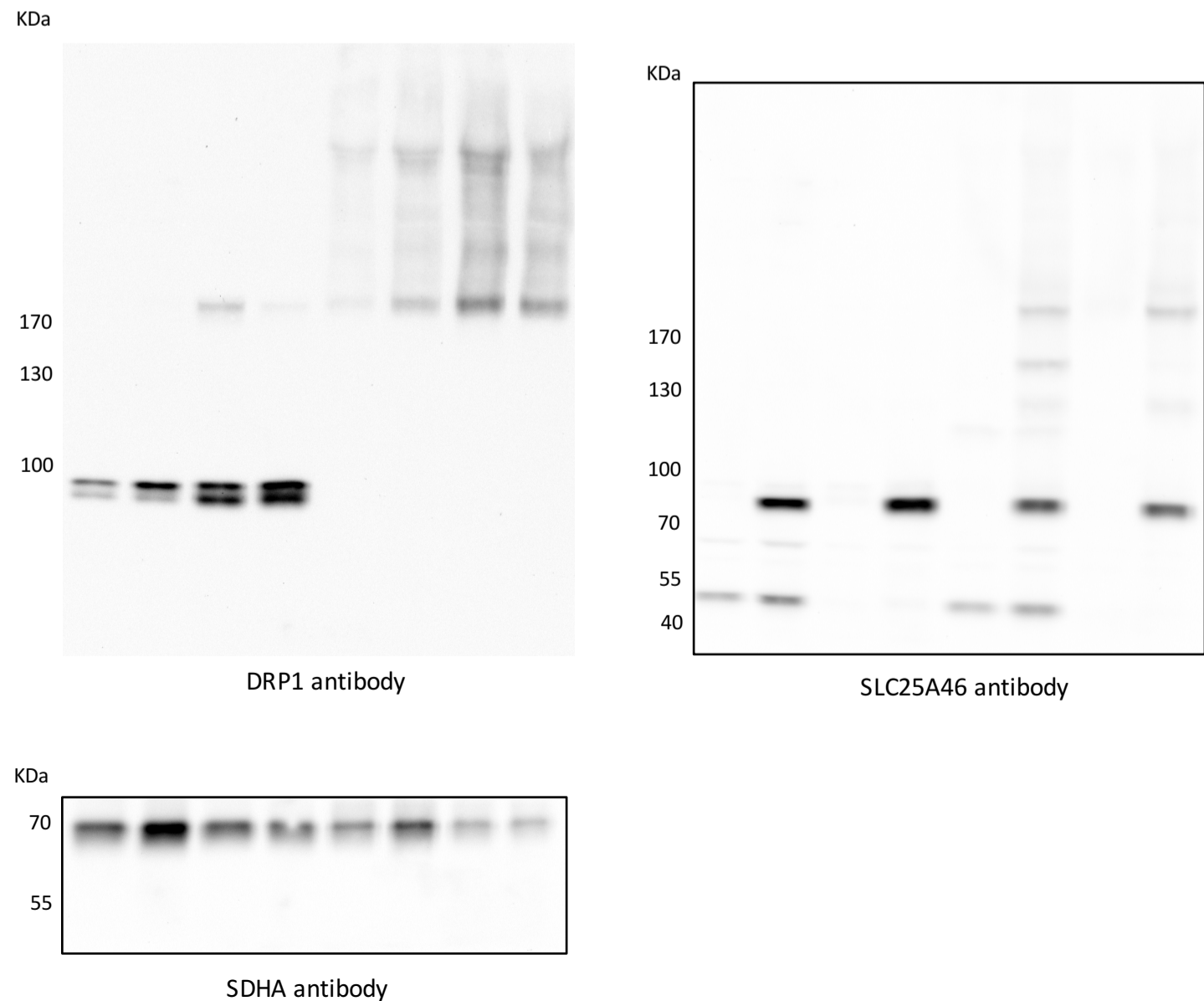

Supplement: Supplementary file 7 — Source Data for Figure 3 [file EMMM-8-1019-s005.pdf]

# Figure 4 raw data

Figure 4D

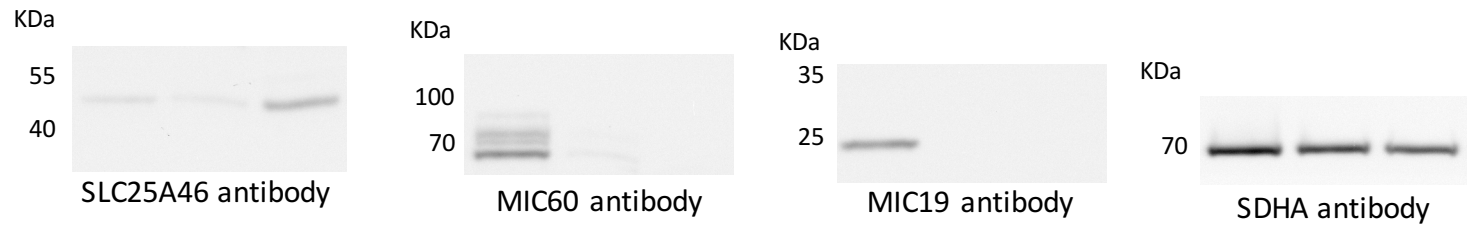

Figure 4E

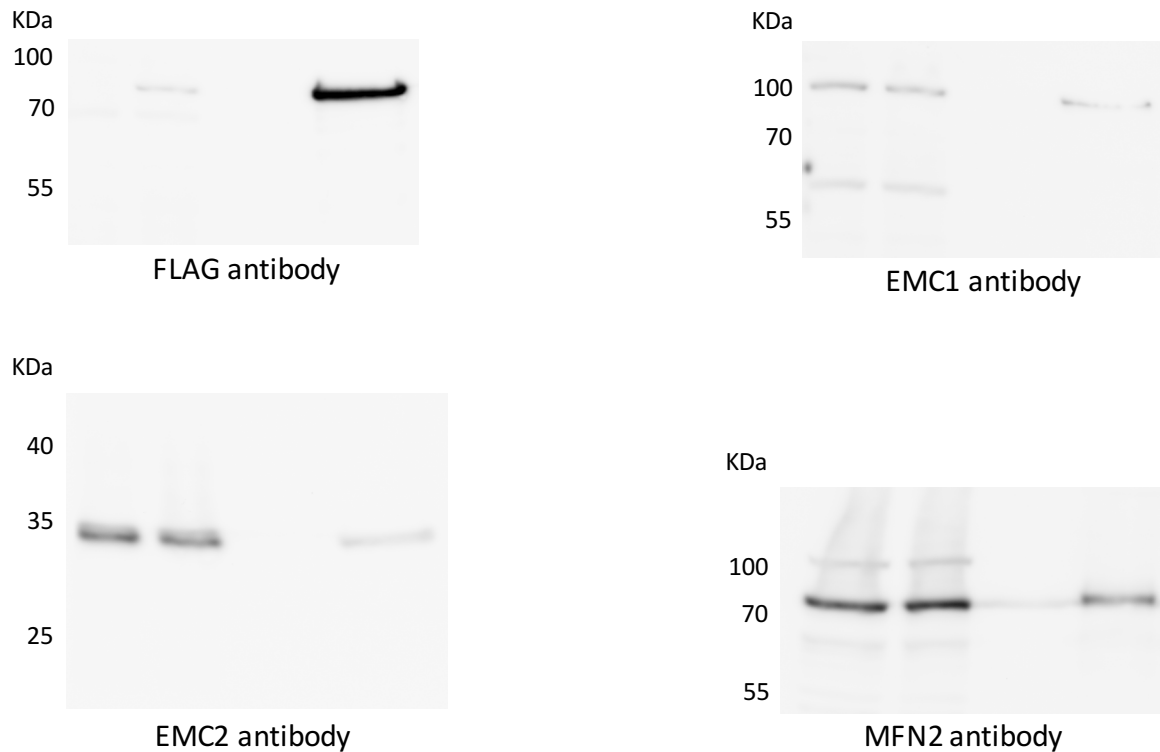

Supplement: Supplementary file 8 — Source Data for Figure 4 [file EMMM-8-1019-s006.pdf]

Figure 7 raw data

Figure 7E

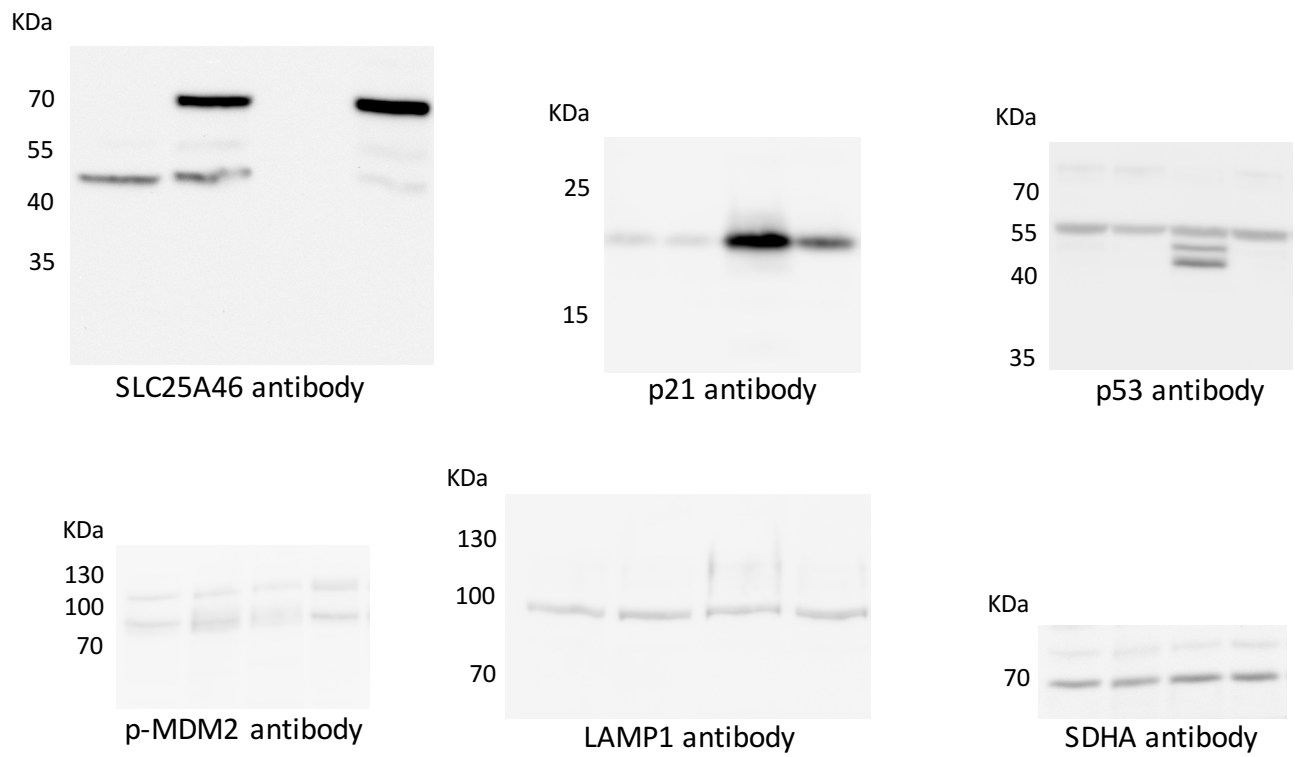

Figure 7I

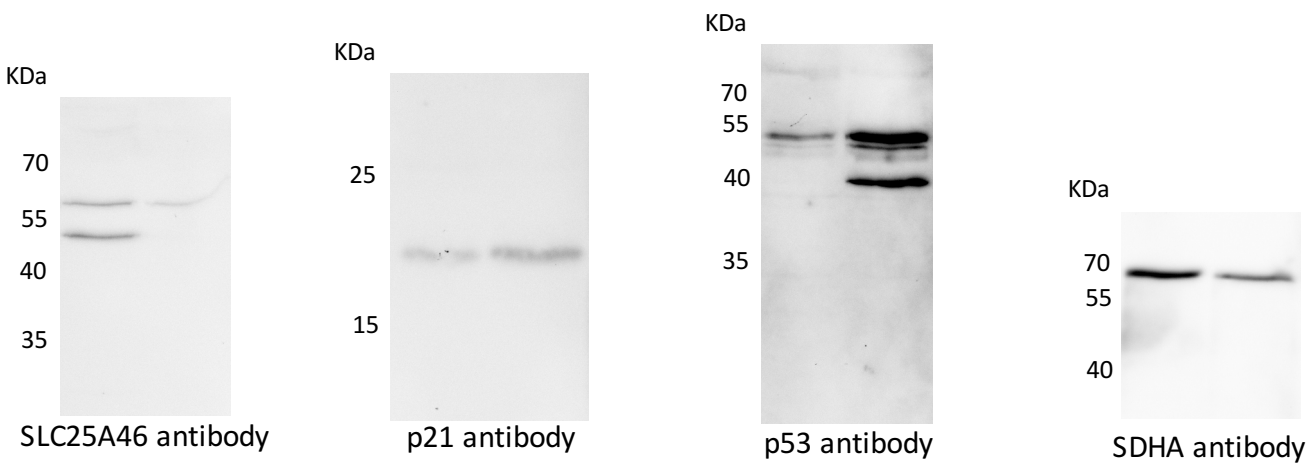

Figure 7K

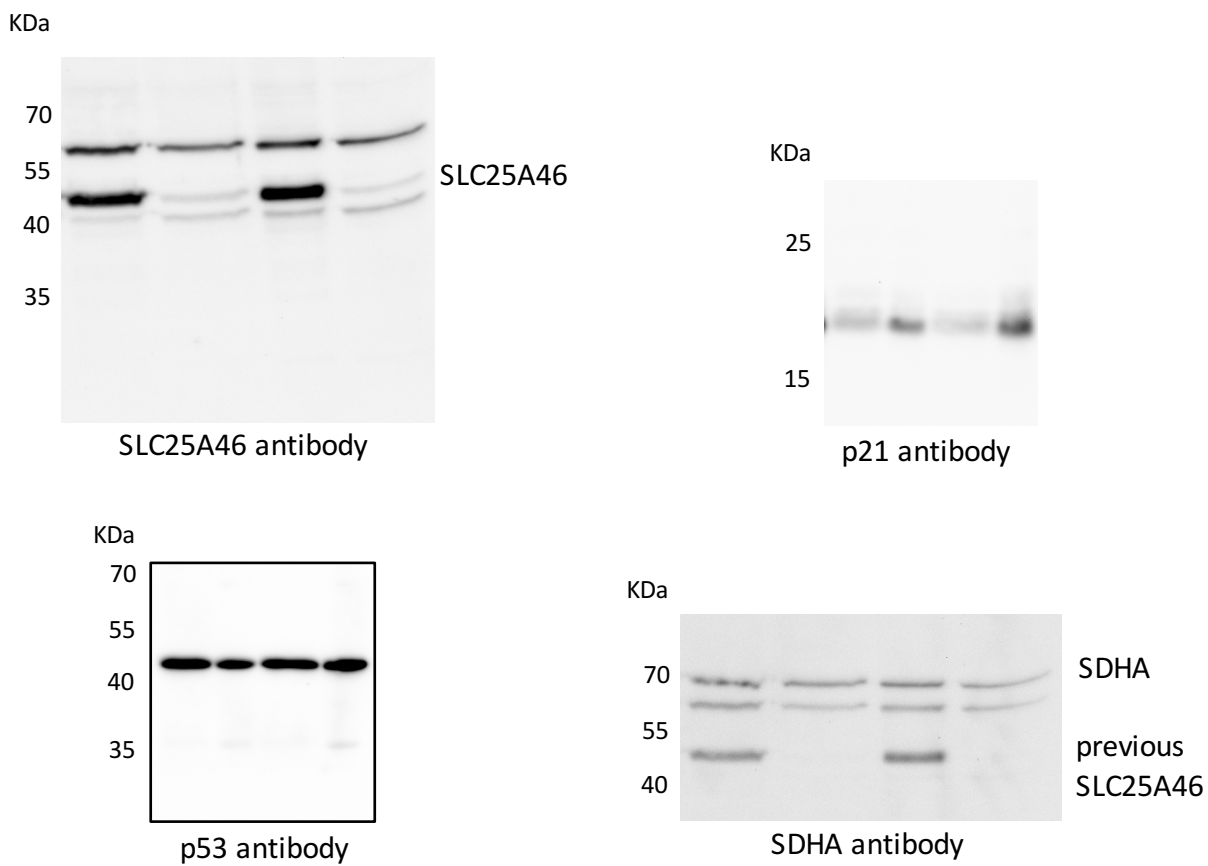

Supplement: Supplementary file 9 — Source Data for Figure 7 [file EMMM-8-1019-s007.pdf]
